# Noncontiguous T cell epitopes in autoimmune diabetes: From mice to men and back again

**DOI:** 10.1016/j.jbc.2021.100827

**Published:** 2021-05-24

**Authors:** Nitin Amdare, Anthony W. Purcell, Teresa P. DiLorenzo

**Affiliations:** 1 Department of Microbiology and Immunology, Albert Einstein College of Medicine, Bronx, New York, USA; 2 Infection and Immunity Program and Department of Biochemistry and Molecular Biology, Biomedicine Discovery Institute, Monash University, Clayton, Victoria, Australia; 3 Division of Endocrinology, Department of Medicine, Albert Einstein College of Medicine, Bronx, New York, USA; 4 Einstein-Mount Sinai Diabetes Research Center, Albert Einstein College of Medicine, Bronx, New York, USA; 5 The Fleischer Institute for Diabetes and Metabolism, Albert Einstein College of Medicine, Bronx, New York, USA

**Keywords:** antigen, diabetes, epitope mapping, peptides, T cell, APC, antigen-presenting cell, HIP, hybrid insulin peptide, IEDB, Immune Epitope Database, MHC, major histocompatibility complex, NOD, nonobese diabetic, PaLN, pancreatic lymph node, T1D, type 1 diabetes, TCR, T cell receptor

## Abstract

Type 1 diabetes (T1D) is a T cell–mediated autoimmune disease that affects the insulin-producing beta cells of the pancreatic islets. The nonobese diabetic mouse is a widely studied spontaneous model of the disease that has contributed greatly to our understanding of T1D pathogenesis. This is especially true in the case of antigen discovery. Upon review of existing knowledge concerning the antigens and peptide epitopes that are recognized by T cells in this model, good concordance is observed between mouse and human antigens. A fascinating recent illustration of the contribution of the nonobese diabetic mouse in the area of epitope identification is the discovery of noncontiguous CD4^+^ T cell epitopes. This novel epitope class is characterized by the linkage of an insulin-derived peptide to, most commonly, a fragment of a natural cleavage product of another beta cell secretory granule constituent. These so-called hybrid insulin peptides are also recognized by T cells in patients with T1D, although the precise mechanism for their generation has yet to be defined and is the subject of active investigation. Although evidence from the tumor immunology arena documented the existence of noncontiguous CD8^+^ T cell epitopes, generated by proteasome-mediated peptide splicing involving transpeptidation, such CD8^+^ T cell epitopes were thought to be a rare immunological curiosity. However, recent advances in bioinformatics and mass spectrometry have challenged this view. These developments, coupled with the discovery of hybrid insulin peptides, have spurred a search for noncontiguous CD8^+^ T cell epitopes in T1D, an exciting frontier area still in its infancy.

Beta cells in the pancreatic islets of Langerhans synthesize and secrete insulin, a hormone required for glucose utilization and homeostasis. In autoimmune diabetes, also known as type 1 diabetes (T1D), beta cells are destroyed by T cells that have been activated by islet-derived peptides bound to major histocompatibility complex (MHC) molecules, either displayed by the beta cells themselves or by professional antigen-presenting cells (APCs) (1). Consistent with the presence of CD4^+^ and CD8^+^ T cells specific for beta cell–derived peptides in the islets of donors with T1D (2), both T cell subsets are believed to participate in beta cell elimination. Based on studies in rodent models, T cells likely employ a variety of mechanisms to achieve this end, including Fas-mediated apoptosis and the release of effector molecules such as perforin, granzyme, and the cytokines interferon-γ and tumor necrosis factor-α (3, 4). In the absence of a sufficient beta cell mass, exogenous insulin becomes necessary for survival.

T1D is a complex disease with both genetic and environmental components (5). Polymorphisms in dozens of genes contribute to disease susceptibility or resistance (6). The majority are expressed by cells of the immune system or by the pancreatic beta cells themselves, reflecting a complicated interplay between autoreactive T cells and beta cells. Environmental triggers (*e.g.*, viral infection or dietary components) that initiate an often protracted, and initially asymptomatic, autoimmune process in genetically susceptible individuals are assumed, but remain ill-defined (7). Adding to the complexity is the finding of serum autoantibodies to beta cell proteins, often years before the onset of clinical symptoms (8). Although autoantibodies are of great utility in predicting individuals who will develop T1D, a pathogenic role for the autoantibodies has not been established, and the disease is viewed as being mediated by T cells rather than by antibodies.

Given the essential role of T cell epitopes in the pathogenesis of T1D, it is unsurprising that multiple benefits have been derived from their identification, and others can be readily envisioned. Knowledge regarding the T cell epitopes in T1D has provided critical insights into the mechanistic basis of the disease process. For example, it was once satisfying to believe that patients with T1D would harbor T cells specific for beta cell antigens, whereas healthy controls would be devoid of them, having been successfully purged of autoreactive T cells by the central tolerance mechanism of thymic negative selection. However, the identification of T cell epitopes in T1D now allows T cells specific for beta cell antigens to be quantitatively and functionally assessed (albeit thus far for research purposes only), leading to the important realization that CD4^+^ and CD8^+^ T cells reactive to beta cell peptides are present in both health and disease (9, 10). Yet, differences in T cell numbers and/or function are often noted when the two states are compared (11), suggesting the potential utility of antigen-specific T cell assays for immune monitoring, *e.g.*, in disease prevention and reversal trials, or as diagnostic tools. The promise and feasibility of T cell–based assays in a clinical setting is exemplified by the interferon-γ release assays that are currently used in the diagnosis of latent *Mycobacterium tuberculosis* (*Mtb*) infection (12). In these assays, peripheral blood cells are exposed to peptides derived from known *Mtb* antigens, and *Mtb*-specific T cells are detected by the interferon-γ they release in response to recognition of their cognate epitopes. Finally, in addition to representing important components of a future clinical assay to detect beta cell–specific T cells, T cell epitopes are also being explored in clinical trials as preventive or therapeutic agents for T1D (13).

With the above goals and opportunities in mind, discovery of T cell epitopes in T1D continues to be an active area of investigation, and the known peptides recognized by T1D-associated T cells in humans have recently been compiled and evaluated (11). Although the majority of the epitopes identified to date are conventional peptides, T1D-associated T cell epitopes may also be posttranslationally modified or otherwise unconventional (11) (Fig. 1). In view of the known clinical importance of an immune response to posttranslationally modified peptides in rheumatoid arthritis (14) and celiac disease (15), there is currently considerable interest in unconventional epitopes in T1D as well. The collection of biochemical processes that create unconventional T cell epitopes in T1D (Fig. 1) includes disulfide bond formation (16), deamidation (2, 17, 18, 19), citrullination (2, 17, 18, 20, 21), phosphorylation (21), alternative open reading frame usage (22), and translation of alternatively spliced RNA transcripts (23, 24). The formation of noncontiguous T cell epitopes, first revealed by the nonobese diabetic (NOD) mouse model of autoimmune diabetes (25), is a fascinating recent addition to this list that has generated enormous excitement and spawned new avenues of research for T1D investigators (2, 23, 24, 25, 26, 27, 28, 29).

**Figure 1:**
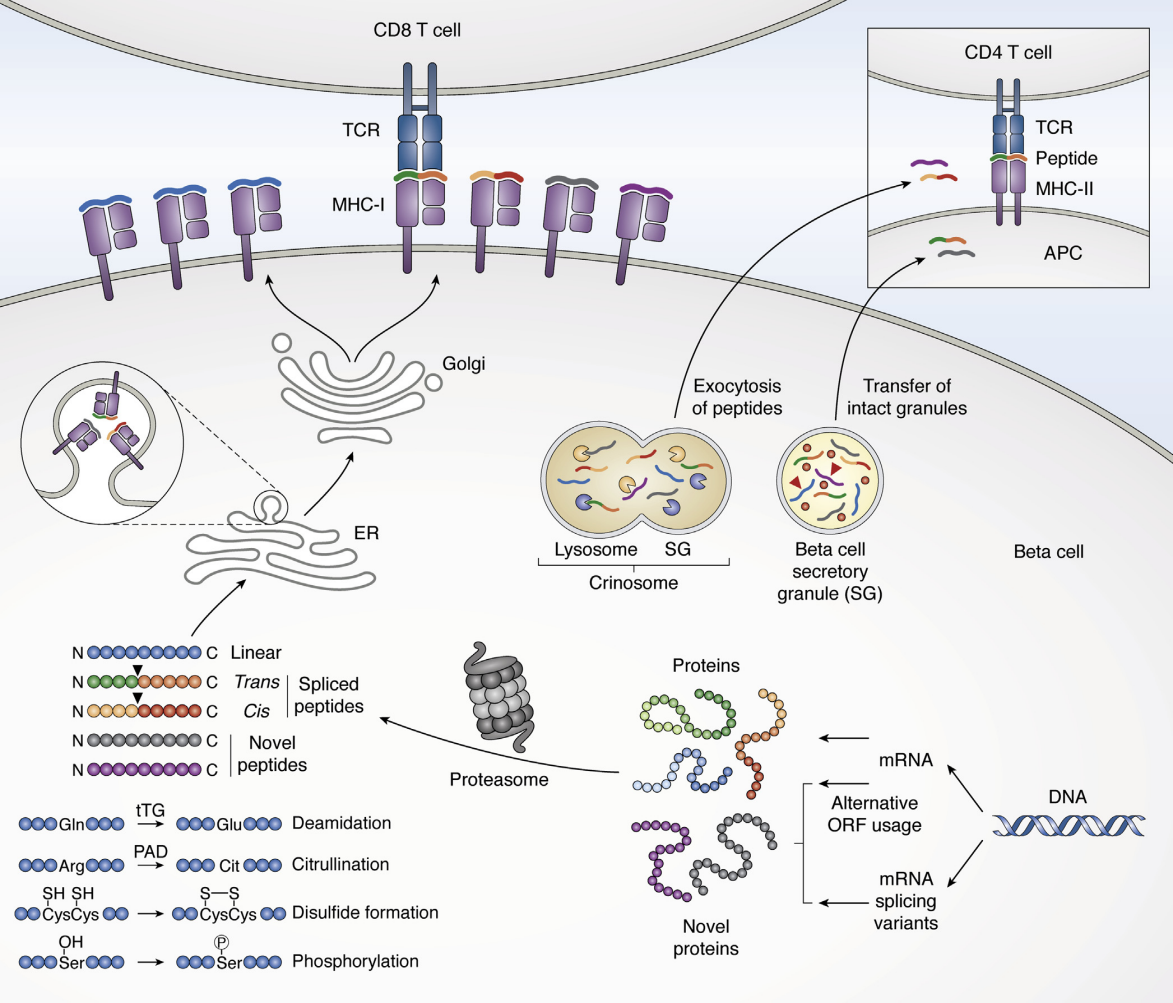
**Unconventional T cell epitopes in T1D**. Besides the formation of noncontiguous T cell epitopes, the focus of this review, a number of other processes create unconventional T cell epitopes in T1D. Alternative open reading frame (ORF) usage and translation of alternatively spliced RNA transcripts can both lead to the formation of novel proteins. The proteasome processes both novel and standard proteins for presentation on class I MHC molecules. It also participates in the formation of noncontiguous CD8^+^ T cell epitopes (*cis*- and *trans*-spliced peptides; *black arrows* denote the border between the two peptide segments). Noncontiguous CD4^+^ T cell epitopes (hybrid insulin peptides, or HIPs; *dual-colored*) likely form in the beta cell secretory granules and crinosomes. For presentation of HIPs on class II MHC molecules, islet APCs can acquire intact secretory granules, with their HIPs, from beta cells, while exocytosed crinosome peptides can be released from beta cells into the circulation and captured by APCs in peripheral lymphoid organs and blood. Deamidation, citrullination, disulfide bond formation, and phosphorylation can also contribute to the formation of unconventional T cell epitopes in T1D (*lower left*). Their exact cellular and subcellular origins have not been fully elucidated, although deamidation by tissue transglutaminase (tTG) and citrullination by protein-arginine deiminase (PAD) are thought to occur in both beta cells and APCs (97, 101, 102). Readers are referred to the text for relevant additional citations. APC, antigen-presenting cell; T1D, type 1 diabetes.

In this review, noncontiguous epitopes in autoimmune diabetes are discussed from a historical perspective. This young yet burgeoning area of research is summarized, and the prospects and challenges that it presents are discussed. Of importance, to facilitate future pioneering discoveries in NOD mice, a long overdue summary of the conventional islet peptides recognized by T cells in this model system is also provided, accompanied by an analysis that further validates NOD mice as an important tool for the gathering of knowledge relevant to human disease.

## The NOD mouse

The primary rodent model used for studying T1D is the NOD mouse (30). First described by Makino and colleagues in 1980, the NOD mouse distinguishes itself from most other murine autoimmunity models in that disease development is spontaneous, requiring no experimental administration of disease-inciting antigens (31). NOD mice and patients with T1D both develop lymphocytic infiltration of their islets (insulitis) and subsequent beta cell destruction mediated by T cells specific for beta cell antigens (32, 33, 34, 35). Multiple genetic loci (referred to as *Idd* in mice and *IDDM* in humans) contribute to disease susceptibility in both NOD mice and patients with T1D (6, 36). Of note, in a number of cases, an *Idd* locus is syntenic with an *IDDM* one. This suggests that common pathogenic mechanisms are responsible for T1D development in both mice and humans. Indeed, in both organisms, the strongest disease link is with the occurrence of particular MHC class II alleles. Furthermore, the sole NOD class II MHC molecule H2-A^g7^ and the human predisposing molecule HLA-DQ8 (a dimer of alpha chain DQA1∗03:01 and beta chain DQB1∗03:02) are structurally related and present similar peptides to CD4^+^ helper T cells (37). These peptides are apparently critical for the initiation and/or propagation of T cell–mediated beta cell injury. The expression of certain MHC class I molecules also contributes to disease susceptibility in both NOD mice and humans (38, 39, 40, 41, 42), again presumably due to their presentation of essential disease-related beta cell peptides to cytotoxic CD8^+^ T cells. These findings are consistent with the detection of beta cell–specific CD4^+^ and CD8^+^ T cells in both species (1). Additional examples of pathogenic mechanisms shared between NOD mice and T1D-susceptible humans are alterations in the T cell–inhibitory cytotoxic T lymphocyte-associated-4 (CTLA-4) pathway (43) and diminished function of CD4^+^CD25^+^ regulatory T cells, essential for peripheral tolerance, due to gene variants affecting interleukin 2 signaling (44, 45, 46). Thus, standard NOD mice have considerable strengths as a preclinical model.

**Figure 2:**
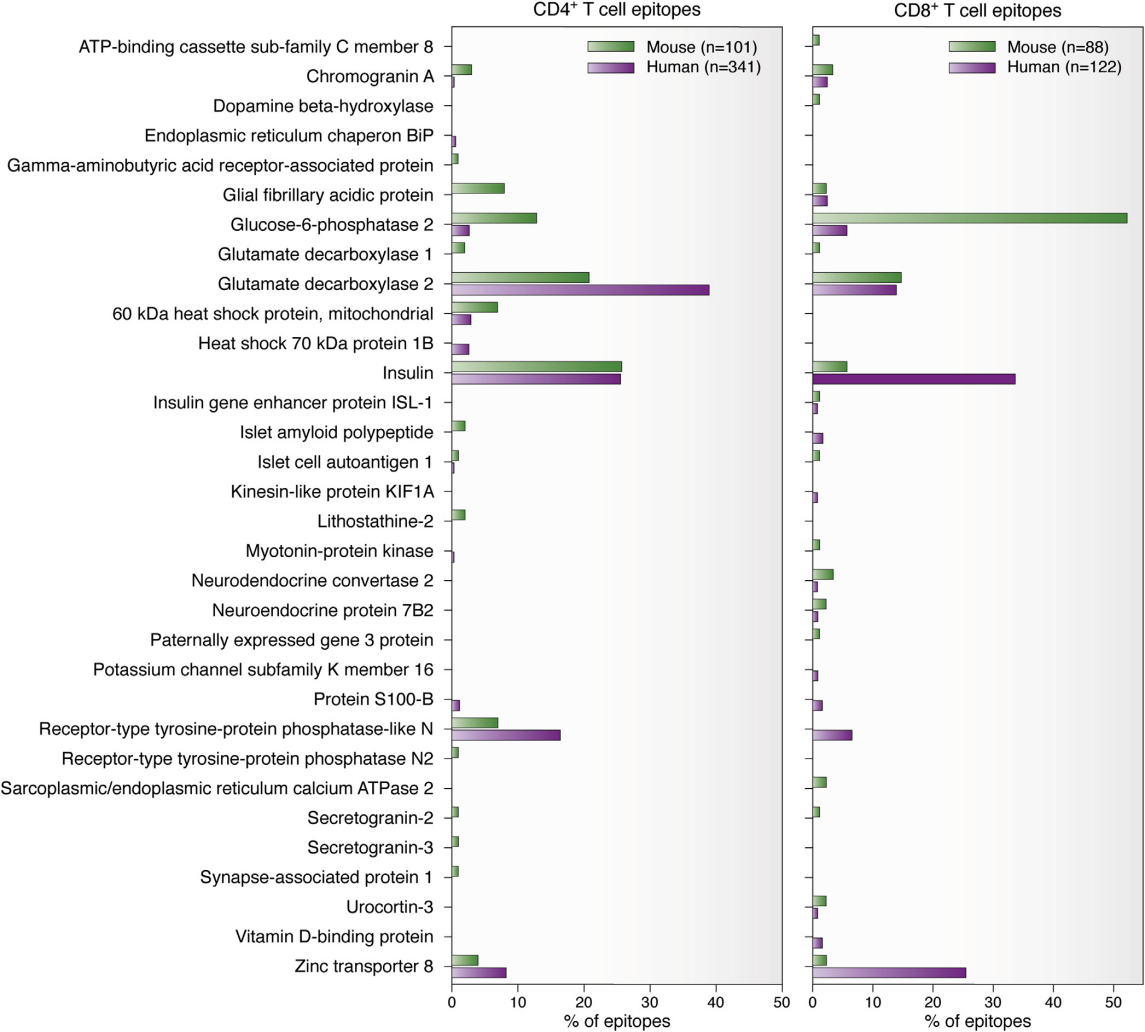
**Demonstration of the utility of the NOD mouse model in the identification of human-relevant islet antigens.** The islet sources of the conventional T cell epitopes in NOD mice (Tables 1 and 2) and humans (11) are listed on the *y*-axis in alphabetical order according to their UniProt consortium names (www.uniprot.org) (103). For NOD mice, “Insulin” includes both Insulin-1 and Insulin-2 epitopes. The percent of all CD4^+^ (*left*) or CD8^+^ (*right*) T cell epitopes that derive from a given protein in each species are plotted on the *x*-axis; “n” indicates the total number of epitopes included in each of the sets. This analysis reveals good concordance between mouse and human antigens for both CD4^+^ and CD8^+^ T cells, as most of the islet proteins that are sources of T cell epitopes in humans also contribute epitopes in NOD mice.

Although imperfections of the NOD mouse model have been noted by others (47), it is nonetheless true that much of what we now understand about the pathogenesis of T1D has been learned from investigation of the NOD mouse and its genetically altered derivative strains. One prime illustration of this is the utility of the NOD mouse model in the identification of human-relevant beta cell antigens, with the discovery of glucose-6-phosphatase 2 (48) and chromogranin-A being two such examples (49). To rigorously demonstrate this point, we compiled a list of the conventional islet-derived T cell epitopes that have been reported in NOD mice. Previous reviews on this topic (50, 51), now greater than a decade old, were used as the foundation, with substantial updates drawn from searches of the Immune Epitope Database (www.iedb.org) (52) and PubMed, using strategies analogous to those described for human T1D-relevant epitopes (11). These efforts yielded comprehensive lists of conventional islet-derived T cell epitopes recognized by CD4^+^ and CD8^+^ T cells in NOD mice (Tables 1 and 2, respectively). The lists include the Immune Epitope Database number for each epitope, as well as whether T cell responses were observed spontaneously (with the T cell source noted) or subsequent to peptide or protein immunization. The CD4^+^ T cell epitopes were derived from 18 proteins (Table 1), and the CD8^+^ T cell epitopes from 19 (Table 2), with ten proteins (bolded in Tables 1 and 2) contributing both CD4^+^ and CD8^+^ T cell epitopes. Taken together, conventional peptides originating from 27 discrete proteins were found to be T cell epitopes in NOD mice. By consulting a recent compilation of the islet-derived antigens recognized by T cells in humans (11), it was determined that 56% of the antigenic mouse proteins are also sources of conventional T cell epitopes in humans (15/27, with the proteins encoded by *Ins2* and *INS* considered a match for the purpose of this calculation). Similarly, 71% (15/21) of the islet proteins that are sources of conventional T cell epitopes in humans (11) also yield T cell epitopes in NOD mice, with good concordance seen for both CD4^+^ and CD8^+^ T cell epitopes (Fig. 2). This analysis emphasizes the utility of NOD mice in the identification of islet antigens that translate to human T1D.

**Table 1:** CD4^+^ T cell epitopes for islet antigens in NOD mice

Protein (*gene*)	Position	Sequence	IEDB epitope identifier	MHC	T cell source			Reference
					Peptide immunization	Protein immunization	Spontaneous	
**Chromogranin-A** (*ChgA*)	29–42	DTKVMKCVLEVISD	142130	A^g7^	Yes		Islets, PaLN	(104, 105)
	358–371	WSRMDQLAKELTAE	131150	A^g7^	Yes		Pooled islets and PaLN; spleen	(49, 60, 104, 106)
	407–423	RPSSREDSVEARSDFEE	224951	A^g7^	Yes			(107)
Gamma-aminobutyric acid receptor–associated protein (*Gabarap*)	29–45	VPVIVEKAPKARIGDLD	225099	A^g7^	Yes		PaLN	(107)
**Glial fibrillary acidic protein** (*Gfap*)	51–65	LAGALNAGFKETRAS	106588	A^g7^		Yes		(108)
	96–110	AELNQLRAKEPTKLA	106229	A^g7^	Yes	Yes	Spleen	(108)
	106–120	PTKLADVYQAELREL	106718	A^g7^		Yes		(108)
	116–130	ELRELRLRLDQLTAN	106393	A^g7^	Yes	Yes	Spleen	(108)
	206–220	RELREQLAQQQVHVE	106769	A^g7^		Yes		(108)
	216–230	QVHVEMDVAKPDLTA	106753	A^g7^	Yes	Yes	Spleen	(108)
	241–255	AVATSNMQETEEWYR	106285	A^g7^		Yes		(108)
	331–345	EGQSLKEEMARHLQE	106382	A^g7^		Yes		(108)
**Glucose-6-phosphatase 2** (*G6pc2*)	4–22	LHRSGVLIIHHLQEDYRTY	104553	A^g7^	Yes		Spleen	(109)
	17–34	EDYRTYYGFLNFMSNVGD	178720	A^g7^		Yes		(110)
	55–72	TKMIWVAVIGDWFNLIFK	179568	A^g7^		Yes	PaLN	(110)
	123–145	WYVMVTAALSYTISRMEESSVTL	104688	A^g7^	Yes		Spleen	(109)
	125–142	VMVTAALSYTISRMEESS	179653	A^g7^		Yes		(110)
	128–145	TAALSYTISRMEESSVTL	104646	A^g7^	Yes		Spleen	(109)
	141–156	SSVTLHRLTWSFLWSV	179534	A^g7^		Yes		(110)
	179–196	VILGVIGGMLVAEAFEHT	179631	A^g7^		Yes		(110)
	195–214	HTPGVHMASLSVYLKTNVFL	104519	A^g7^	Yes		Spleen	(109)
	241–256	KWCANPDWIHIDSTPF	179108	A^g7^		Yes		(110)
	271–288	FAINSEMFLRSCQGENGT	178790	A^g7^		Yes	PaLN	(110)
	301–318	LTTMQLYRFIKIPTHAEP	179219	A^g7^		Yes	PaLN	(110)
	309–326	FIKIPTHAEPLFYLLSFC	178817	A^g7^		Yes	PaLN	(110)
**Glutamate decarboxylase 1** (*Gad1*)	29–48	DTWCGVAHGCTRKLGLKICG	104757	A^g7^	Yes		Spleen	(111)
	44–62	LKICGFLQRTNSLEEKSRL	104883	A^g7^	Yes		Spleen	(111)
**Glutamate decarboxylase 2** (*Gad2*)	118–128	LLQYVVKSFDR	104044	A^g7^	Yes			(112)
	202–221	TNMFTYEIAPVFVLLEYVTL	105004	A^g7^	Yes	Yes	Spleen	(111)
	206–220	TYEIAPVFVLLEYVT	67328	A^g7^	Yes	Yes		(113, 114)
	208–217	EIAPVFVLLE	103138	A^g7^	Yes			(115)
	217–236	EYVTLKKMREIIGWPGGSGD	104481	A^g7^	Yes	Yes	Islets, PaLN, spleen	(111, 116)
	221–235	LKKMREIIGWPGGSG	102615	A^g7^	Yes	Yes		(114, 117)
	232–251	GGSGDGIFSPGGAISNMYAM	105216	A^g7^			Spleen	(116)
	247–266	NMYAMLIARYKMSPEVKEKG	102680	A^g7^	Yes		Spleen	(118, 119)
	268–278	AAVPRLIAFTS	103782	A^g7^	Yes			(112)
	286–300	KKGAAALGIGTDSVI	102562	A^g7^	Yes	Yes		(114, 120)
	290–309	AALGIGTDSVILIKCDERGK	104403	A^g7^	Yes		Islets, PaLN, spleen	(116, 121)
	316–335	ERRILEVKQKGFVPFLVSAT	104477	A^g7^			Spleen	(122)
	401–415	PLQCSALLVREEGLM	104599	A^g7^		Yes		(114)
	509–524	VPPSLRTLEDNEERMS	104672	A^g7^			Spleen	(123)
	509–528	VPPSLRTLEDNEERMSRLSK	102913	A^g7^	Yes	Yes	Spleen	(118, 119, 124)
	524–538	SRLSKVAPVIKARMM	60728	A^g7^			Spleen	(125, 126)
	524–543	SRLSKVAPVIKARMMEYGTT	102085	A^g7^	Yes	Yes	Spleen	(113, 118, 127)
	530–543	APVIKARMMEYGTT	103810	A^g7^	Yes		Spleen	(126)
	531–545	PVIKARMMEYGTTMV	104605	A^g7^			Spleen	(122)
	561–575	ISNPAATHQDIDFLI	104845	A^g7^		Yes	Spleen	(114, 116)
	571–585	IDFLIEEIERLGQDL	102525	A^g7^		Yes		(128)
60-kDa heat shock protein, mitochondrial (*Hspd1*)	76–95	DGVTVAKSIDLKDKYKNIGA	102353	A^g7^	Yes			(129)
	166–185	EEIAQVATISANGDKDIGNI	102381	A^g7^	Yes			(129)
	195–214	RKGVITVKDGKTLNDELEII	102765	A^g7^	Yes			(129)
	361–380	KGDKAHIEKRIQEITEQLDI	103303	A^g7^	Yes			(129)
	437–460	VLGGGCALLRCIPALDSLKPANED	105025	A^g7^			Spleen	(130)
	526–545	RTALLDAAGVASLLTTAEAV	103572	A^g7^	Yes			(129)
	541–560	TAEAVVTEIPKEEKDPGMGA	103630	A^g7^	Yes			(129)
**Insulin-1** (*Ins1*)	7–23	FLPLLALLALWEPKPTQ	105786	A^g7^	Yes			(131)
	7–24	FLPLLALLALWEPKPTQA	133571	A^g7^	Yes		Islets	(132)
	20–35	KPTQAFVKQHLCGPHL	105906	A^g7^	Yes			(131)
	33–47	PHLVEALYLVCGERG	104594	A^g7^	Yes		Islets	(131, 133)
	34–53	HLVEALYLVCGERGFFYTPK	105241	A^g7^	Yes			(134)
	36–44	VEALYLVCG	104666	A^g7^			Pooled islets and PaLN	(60)
	37–47	EALYLVCGERG	104759	A^g7^			Islets	(135)
	57–85	EVEDPQVEQLELGGSPGDLQTLA LEVARQ		A^g7^			Pooled islets and PaLN	(60)
	61–85	PQVEQLELGGSPGDLQTLALEV ARQ		A^g7^			Pooled islets and PaLN	(60)
	71–86	SPGDLQTLALEVARQK	106117	A^g7^	Yes		Islets	(131)
	71–88	SPGDLQTLALEVARQKRG	104984	A^g7^	Yes		PaLN	(136)
	73–87	GDLQTLALEVARQKR		A^g7^	Yes			(60)
	75–85	LQTLALEVARQ		A^g7^			Pooled islets and PaLN	(60)
	77–92	TLALEVARQKRGIVDQ	106141	A^g7^	Yes			(131)
	94–108	CTSICSLYQLENYCN	102341	A^g7^			Islets	(137)
**Insulin-2** (*Ins2*)	14–30	LFLWESHPTQAFVKQHL	105927	A^g7^	Yes			(131)
	20–35	HPTQAFVKQHLCGSHL	105866	A^g7^	Yes			(138)
	26–41	VKQHLCGSHLVEALYL	106166	A^g7^			Islets	(131)
	33–40	SHLVEALY	104977	A^g7^			Islets	(135)
	33–47	SHLVEALYLVCGERG	58388	A^g7^	Yes		Islets, spleen	(131, 133, 139)
	36–44	VEALYLVCG	104666	A^g7^			Pooled islets and PaLN	(60)
	37–47	EALYLVCGERG	104759	A^g7^			Islets	(135)
	48–57	FFYTPMSRRE	106415	A^g7^		Yes	PaLN, spleen	(140)
	48–60	FFYTPMSRREVED	102425	A^g7^			Islets	(141)
	71–88	GPGAGDLQTLALEVAQQK	105842	A^g7^	Yes			(131)
	96–110	CTSICSLYQLENYCN	102341	A^g7^			Islets	(137)
Islet amyloid polypeptide (*Iapp*)	38–57	KCNTATCATQRLANFLVRSS	189990	A^g7^			Islets	(142, 143)
	78–90	NAARDPNRESLDF		A^g7^			Pooled islets and PaLN	(60)
**Islet cell autoantigen 1** (*Ica1*)	35–46	AFIKATGKKEDE	104413	A^g7^	Yes	Yes	Spleen	(144)
Lithostathine-2 (*Reg2*)	44–63	PEGANAYGSYCYYLIEDRLT	226248	A^g7^	Yes			(145)
	48–64	NAYGSYCYYLIEDRLTW	226242	A^g7^			PaLN	(145)
Receptor-type tyrosine-protein phosphatase-like N (*Ptprn*)	676–693	PSWCEEPAQANMDISTGH	104939	A^g7^	Yes			(146)
	691–708	TGHMILAYMEDHLRNRDR	104999	A^g7^	Yes			(146)
	706–723	RDRLAKEWQALCAYQAEP	104959	A^g7^	Yes			(146)
	751–768	IKLKVESSPSRSDYINAS	104838	A^g7^	Yes			(146)
	766–783	NASPIIEHDPRMPAYIAT	104909	A^g7^	Yes			(146)
	781–798	IATQGPLSHTIADFWQMV	104836	A^g7^	Yes			(146)
	961–979	FALTAVAEEVNAILKALPQ	104772	A^g7^	Yes			(146)
Receptor-type tyrosine-protein phosphatase N2 (*Ptprn2*)	636–655	KLSGLGADPSADATEAYQEL	104857	A^g7^	Yes			(147)
**Secretogranin-2** (*Scg2*)	234–248	DVYKTNNIAYEDVVG	224671	A^g7^	Yes			(107)
Secretogranin-3 (*Scg3*)	229–244	IPEKVTPVAAVQDGFT	224790	A^g7^	Yes		PaLN	(107)
Synapse-associated protein 1 (*Syap1*)	262–279	TPPVVIKSQLKSQEDEEE	225035	A^g7^			PaLN	(107)
**Zinc transporter 8** (*Slc30a8*)	212–225	SVRAAFVHALGDVF	232569	A^g7^	Yes			(148)
	313–326	ILSVHVATAASQDS	110292	A^g7^	Yes		Pooled islets and PaLN	(60, 148)
	330–344	RTGIAQALSSFDLHS	232561	A^g7^	Yes	Yes		(148)
	345–359	LTIQIESAADQDPSC	232549	A^g7^	Yes	Yes		(148)

Proteins in bold contribute both CD4^+^ and CD8^+^ T cell epitopes.

Abbreviations: IEDB, Immune Epitope Database; PaLN, pancreatic lymph node.

**Table 2:** CD8^+^ T cell epitopes for islet antigens in NOD mice

Protein (*gene*)	Position	Sequence	IEDB epitope identifier	MHC	T cell source			Reference
					Peptide immunization	Protein immunization	Spontaneous	
ATP-binding cassette subfamily C member 8 (*Abcc8*)	229–237	TYWWMNAFI	1311101	K^d^			Islets	(149)
**Chromogranin-A** (*ChgA*)	36–44	VLEVISDSL	142326	K^d^	Yes		Islets, PaLN	(105)
	265–273	HFHAGYKAI	1311038	K^d^			Islets	(149)
	438–446	QELESLSAI	1311072	K^d^			Islets	(149)
Dopamine beta-hydroxylase (*Dbh*)	233–241	TYWCYITEL	546779	K^d^			PaLN, spleen	(150)
**Glial fibrillary acidic protein** (*Gfap*)	79–87	SYIEKVRFL	106886	K^d^	Yes		Spleen	(108)
	253–261	WYRSKFADL	107000	K^d^	Yes		Spleen	(108)
**Glucose-6-phosphatase 2** (*G6pc2*)	2–10	DFLHRSGVL	105134	K^d^			Islets	(151)
	3–11	FLHRSGVLI	105188	D^b^			Islets	(151)
	18–26	DYRTYYGFL	105157	K^d^			Islets	(151)
	21–29	TYYGFLNFM	105560	K^d^			Islets	(151)
	33–41	GDPRNIFSI	105208	D^b^			Islets	(151)
	41–49	IYFPLWFQL	105257	K^d^			Islets	(151)
	48–56	QLNQNVGTK	105486	D^b^			Islets	(151)
	50–58	NQNVGTKMI	105411	D^b^			Islets	(151)
	66–74	WFNLIFKWI	105587	K^d^			Islets	(151)
	89–97	IYPNHSSPC	105258	K^d^			Islets	(151)
	90–98	YPNHSSPCL	105619	D^b^			Islets	(151)
	114–122	GHAMGSSCV	105217	D^b^			Islets	(151)
	130–138	ALSYTISRM	105099	D^b^			Islets	(151)
	133–141	YTISRMEES	105622	D^b^			Islets	(151)
	136–144	SRMEESSVT	105527	D^b^			Islets	(151)
	137–145	RMEESSVTL	105500	D^b^			Islets	(151)
	140–148	ESSVTLHRL	105171	D^b^			Islets	(151)
	154–162	WSVFWLIQI	105609	D^b^			Islets	(151)
	156–164	VFWLIQISV	105569	K^d^			Islets	(151)
	167–175	SRVFIATHF	105528	D^b^			Islets	(151)
	172–180	ATHFPHQVI	105106	D^b^			Islets	(151)
	173–181	THFPHQVIL	105544	D^b^			Islets	(151)
	193–201	FEHTPGVHM	105181	D^b^			Islets	(151)
	204–212	LSVYLKTNV	105312	K^d^			Islets	(151)
	206–214	VYLKTNVFL	102926	K^d^			Blood, islets, PaLN, spleen	(48, 150, 152)
	219–227	LGFYLLLRL	105290	D^b^			Islets	(151)
	225–233	LRLFGIDLL	105308	D^b^			Islets	(151)
	241–249	KWCANPDWI	105272	D^b^			Islets	(151)
	243–251	CANPDWIHI	105117	D^b^			Islets	(151)
	258–266	GLVRNLGVL	105229	D^b^			Islets	(151)
	269–277	LGFAINSEM	105289	D^b^			Islets	(151)
	270–278	GFAINSEMF	105210	D^b^			Islets	(151)
	271–279	FAINSEMFL	105175	D^b^			Islets	(151)
	282–290	CQGENGTKP	105123	D^b^			Islets	(151)
	287–295	GTKPSFRLL	105233	D^b^			Islets	(151)
	296–304	CALTSLTTM	105116	D^b^			Islets	(151)
	298–306	LTSLTTMQL	105316	D^b^			Islets	(151)
	299–307	TSLTTMQLY	105554	D^b^			Islets	(151)
	304–312	MQLYRFIKI	105343	D^b^			Islets	(151)
	308–316	RFIKIPTHA	105495	K^d^			Islets	(151)
	311–319	KIPTHAEPL	105262	D^b^			Islets	(151)
	314–322	THAEPLFYL	105543	D^b^			Islets	(151)
	315–323	HAEPLFYLL	105235	K^d^			Islets	(151)
	323–331	LSFCKSASI	105310	D^b^			Islets	(151)
	324–332	SFCKSASIP	105516	K^d^			Islets	(151)
	326–334	CKSASIPLM	105121	D^b^			Islets	(151)
**Glutamate decarboxylase 1** (*Gad1*)	515–524	WYIPQSLRGV	104687	K^d^	Yes			(153)
**Glutamate decarboxylase 2** (*Gad2*)	85–95	GDVNYAFLHAT	103916	K^d^	Yes			(112)
	88–98	NYAFLHATDLL	104162	K^d^	Yes			(112)
	90–98	AFLHATDLL	104414	K^d^		Yes	PaLN, spleen	(154)
	118–128	LLQYVVKSFDR	104044	K^d^	Yes			(112)
	124–134	KSFDRSTKVID	104015	K^d^	Yes			(112)
	136–146	HYPNELLQEYN	103979	K^d^	Yes			(112)
	139–149	NELLQEYNWEL	104145	K^d^	Yes			(112)
	178–186	YFNQLSTGL	104689	K^d^			PaLN, spleen	(150)
	206–214	TYEIAPVFV	104661	K^d^	Yes		Spleen	(155)
	268–278	AAVPRLIAFTS	103782	K^d^	Yes			(112)
	507–516	WFVPPSLRTL	1123568	K^d^	Yes			(156)
	544–554	MVSYQPLGDKV	104138	K^d^	Yes		Spleen	(112)
	546–554	SYQPLGDKV	104294	K^d^	Yes		PaLN, spleen	(154, 155)
**Insulin-1** (*Ins1*)	39–47	LYLVCGERG	102639	K^d^	Yes		Islets, PaLN, spleen	(150, 157, 158)
	101–107	YQLENYC	233116	D^b^			Islets	(71)
**Insulin-2** (*Ins2*)	39–47	LYLVCGERG	102639	K^d^	Yes		Islets, PaLN, spleen	(150, 157, 158)
	49–58	FYTPMSRREV	102453	K^d^	Yes			(159)
	103–109	YQLENYC	233116	D^b^			Islets	(71)
Insulin gene enhancer protein ISL-1 (*Isl1*)	65–74	TYCKRDYIRL	1312049	K^d^			Islets	(23)
**Islet cell autoantigen 1** (*Ica1*)	78–86	LYQKRICFL	546770	K^d^			PaLN	(150)
Myotonin-protein kinase (*Dmpk*)	138–146	FQDENYLYL	104489	D^b^			Islets	(160)
Neuroendocrine convertase 2 (*Pcsk2*)	320–328	GYASSMWTI	1311036	K^d^			Islets	(149)
	341–350	LYDESCSSTL	1312021	K^d^			Islets	(23)
	501–510	RYLEHVQAVI	1312037	K^d^			Islets	(23)
Neuroendocrine protein 7B2 (*Scg5*)	26–35	AYSPRTPDRV	1311981	K^d^			Islets	(23)
	193–201	DNVVAKKSV	1311986	K^d^			Islets	(23)
Paternally expressed gene 3 protein (*Peg3*)	522–530	CKVCGESFL	1311015	K^d^			Islets	(149)
Sarcoplasmic/endoplasmic reticulum calcium ATPase 2 (*Atp2a2*)	688–696	EFLQSFDEI	1311022	K^d^			Islets	(149)
	835–843	RYLAIGCYV	1311083	K^d^			Islets	(149)
**Secretogranin-2** (*Scg2*)	469–477	PYGPGKSRA	1311070	K^d^			Islets	(149)
Urocortin-3 (*Ucn3*)	5–13	TYFLLPLLL	1312050	K^d^			Islets	(23)
	32–40	VFSCLNTAL	1312051	K^d^			Islets	(23)
**Zinc transporter 8** (*Slc30a8*)	158–166	LYLACERLL	546769	K^d^			PaLN, spleen	(150)
	282–290	SYNSVKEII	546777	K^d^			PaLN, spleen	(150)

Proteins in bold contribute both CD4^+^ and CD8^+^ T cell epitopes.

Abbreviations: IEDB, Immune Epitope Database; PaLN, pancreatic lymph node.

## Noncontiguous CD4^+^ T cell epitopes

**Figure 3:**
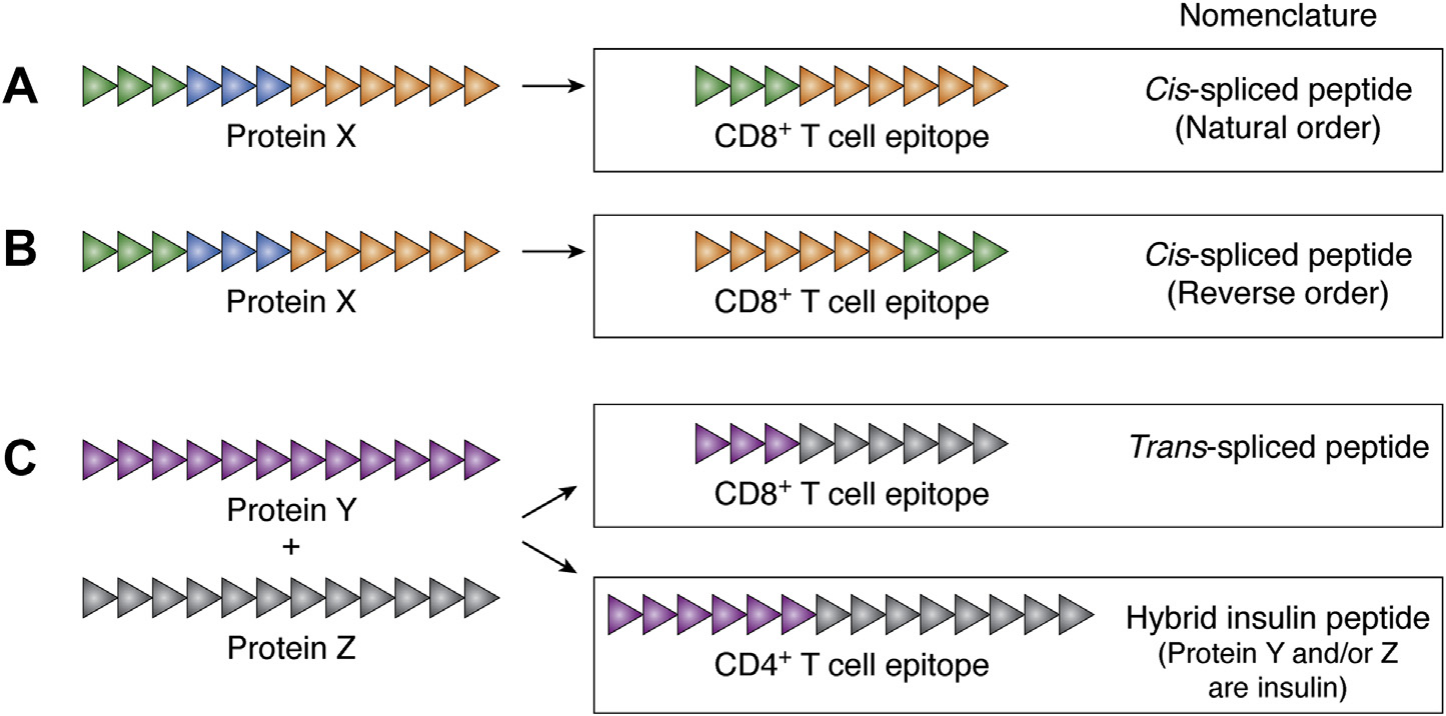
**Nomenclature for noncontiguous T cell epitopes.***A* and *B*, shown at the left are amino acid residues of a segment from hypothetical protein X, where each triangle represents one amino acid. *Green* and *orange* residues denote the peptide fragments that constitute the CD8^+^ T cell epitopes formed by peptide splicing and shown at the right. *Blue* residues denote the missing intervening sequence. The segments of *cis*-spliced peptides can either appear in their natural order (*i.e.*, as they appear in the protein) (*A*) or in reverse order (*B*). *C*, depicted in *purple* and *gray* are hypothetical segments of two proteins, Y and Z. Peptides from two different protein molecules can form CD8^+^ T cell epitopes by *trans*-splicing, in which each protein contributes residues to the resulting peptide epitope (*top right*). In the text, the term “spliced peptide” is reserved for a CD8^+^ T cell epitope or a class I MHC ligand, whereas a CD4^+^ T cell epitope in which the *purple* and/or *gray* segments are derived from insulin (*bottom right*) is referred to as a hybrid insulin peptide.

A recent and paradigm-shifting advance that originated from study of the NOD mouse is the discovery of hybrid insulin peptides, or HIPs, as noncontiguous epitopes for beta cell–reactive CD4^+^ T cells (25). HIPs were first identified as the targets of several long-studied pathogenic CD4^+^ T cell clones from NOD mice (25) and were subsequently shown to be recognized by islet-infiltrating CD4^+^ T cells from patients with T1D (2, 25). In the HIPs that have been reported to date (Table 3), a peptide segment derived from insulin (often including a portion of the normally excised C-peptide) is fused to a second peptide segment almost exclusively derived either from a different insulin secretory granule protein or from a noncontiguous portion of insulin itself (Fig. 3*C*). Although noncontiguous CD4^+^ T cell epitopes had not previously been described in any disease, since 2004 there have been several reports of noncontiguous epitopes generated by the proteasome and recognized by tumor-reactive cytotoxic CD8^+^ T cells isolated from cancer patients (53, 54, 55, 56, 57). Such CD8^+^ T cell epitopes, formed by a process termed “peptide splicing,” are characterized by the covalent linkage of two peptides derived from the same protein (*cis*-spliced peptides) (Fig. 3, *A* and *B*) or different proteins (*trans*-spliced peptides) (Fig. 3*C*). In the case of *cis*-spliced peptides, an intervening protein sequence has been removed, and the two linked segments can either be in their natural order (*i.e.*, as they appear in the protein) (Fig. 3*A*) or in reverse order (Fig. 3*B*). Although HIP epitopes for CD4^+^ T cells may seem at first glance to be analogous to *trans*-spliced tumor epitopes recognized by CD8^+^ T cells (Fig. 3*C*), to date there is no evidence that the proteasome participates in HIP formation. Thus, for clarity, here we reserve the term “spliced peptides” for noncontiguous CD8^+^ T cell epitopes or class I MHC ligands only and do not describe HIPs in this way. This nomenclature, summarized in Figure 3, is consistent with the majority of the literature on this subject.

**Table 3:** Noncontiguous CD4^+^ T cell epitopes for islet antigens in NOD mice and humans

Host	Segment 1			Segment 2			IEDB epitope identifier	MHC	T cell source	Reference
	Protein (*gene*)	Position	Sequence	Protein (*gene*)	Position	Sequence				
Mouse	Insulin-1 (*Ins1*)	75–80	LQTLAL	Chromogranin-A (*ChgA*)	358–362	WSRMD	910154	A^g7^	Blood, islets,	(25, 27)
	Insulin-2 (*Ins2*)	77–82	LQTLAL						PaLN, spleen	
	Insulin-1 (*Ins1*)	75–80	LQTLAL	Islet amyloid polypeptide	78–83	NAARDP	910153	A^g7^	Blood, islets,	(25, 27, 29)
	Insulin-2 (*Ins2*)	77–82	LQTLAL	(*Iapp*)					PaLN, spleen	
Human	Insulin (*INS*)	34–41	HLVEALYL	Secretogranin-1 (*CHGB*)	211–218	EELVARSE	1084801	DR0401	Blood	(26)
	Insulin (*INS*)	42–49	VCGERGFF	Secretogranin-1 (*CHGB*)	211–218	EELVARSE	1086861	DR0401	Blood	(26)
	Insulin (*INS*)	64–70	GQVELGG	Chromogranin-A (*CHGA*)	342–349	WSKMDQLA	1145014	n.d.	Blood	(28)
	Insulin (*INS*)	64–70	GQVELGG	Chromogranin-A (*CHGA*)	358–365	LEGQEEEE	1145010	n.d.	Blood	(28)
	Insulin (*INS*)	64–71	GQVELGGG	Insulin (*INS*)	57–63	EAEDLQV	1310104	n.d.	Blood	(28)
	Insulin (*INS*)	64–71	GQVELGGG	Insulin (*INS*)	90–96	GIVEQCC	583306	n.d.	Blood, islets	(2, 28)
	Insulin (*INS*)	64–71	GQVELGGG	Islet amyloid polypeptide (*IAPP*)	23–29	TPIESHQ	583307	Class II	Islets	(2)
	Insulin (*INS*)	64–71	GQVELGGG	Islet amyloid polypeptide (*IAPP*)	74–80	NAVEVLK	505706	DQ8	Blood, islets	(2, 25, 28)
	Insulin (*INS*)	64–71	GQVELGGG	Pro-neuropeptide Y (*NPY*)	68–74	SSPETLI	505707	DQ8	Blood, islets	(2, 25, 28)
	Insulin (*INS*)	64–71	GQVELGGG	Secretogranin-1 (*CHGB*)	440–446	FLGEGHH	1310105	n.d.	Blood	(28)
	Insulin (*INS*)	76–82	SLQPLAL	Chromogranin-A (*CHGA*)	342–348	WSKMDQL	1169825	n.d.	Blood	(28)
	Insulin (*INS*)	76–82	SLQPLAL	Insulin (*INS*)	57–63	EAEDLQV	1169818	DQ	Blood	(28)
	Insulin (*INS*)	76–82	SLQPLAL	Insulin (*INS*)	90–96	GIVEQCC	1169820	DR	Blood	(28)
	Insulin (*INS*)	76–82	SLQPLAL	Islet amyloid polypeptide (*IAPP*)	23–29	TPIESHQ	1169824	n.d.	Blood	(28)
	Insulin (*INS*)	76–82	SLQPLAL	Islet amyloid polypeptide (*IAPP*)	74–80	NAVEVLK	1169822	DR	Blood	(28)
	Insulin (*INS*)	76–82	SLQPLAL	Secretogranin-1 (*CHGB*)	440–446	FLGEGHH	1169819	n.d.	Blood	(28)
	Insulin (*INS*)	78–85	QPLALEGS	Endoplasmic reticulum chaperone BiP (*HSPA5*)	298–305	ALSSQHQA	1085904	DR0401	Blood	(26)
	Insulin (*INS*)	85–92	SLQKRGIV	Secretogranin-1 (*CHGB*)	211–218	EELVARSE	1086541	DR0401	Blood	(26)
	Insulin (*INS*)	99–106	ICSLYQLE	Insulin (*INS*)	25–32	FVNQHLCG	1084880	DR0401	Blood	(26)
	Insulin (*INS*)	100–107	CSLYQLEN	Neuroendocrine protein 7B2 (*SCG5*)	200–207	SVPHFSDE	1083949	DR0401	Blood	(26)

n.d., not determined, but presumed class II.

Abbreviations: IEDB, Immune Epitope Database; PaLN, pancreatic lymph node.

The line of investigation that ultimately led to the identification of HIPs was initially inspired by studies to identify the peptide recognized by the T cell clone BDC-2.5, one of a set of pathogenic CD4^+^ T cell clones isolated from the lymph nodes and spleens of diabetic NOD mice, with BDC denoting their origin at the Barbara Davis Center for Childhood Diabetes in Colorado (58). When WE14, a natural cleavage product of chromogranin-A, was identified as the epitope recognized by BDC-2.5, it was a puzzling finding, as the peptide was predicted to leave empty the N-terminal portion of the H2-A^g7^ peptide-binding groove (49). Furthermore, mass spectrometry analysis of chromatographic fractions of beta cell extracts revealed that T cell stimulatory activity did not track with the abundance of WE14. Rather, active fractions contained insulin's C-peptide and fragments thereof. This led to the hypothesis, subsequently proven (25), that the epitope for BDC-2.5 is a fusion of the C-terminal portion of a naturally occurring C-peptide fragment (LQTLAL) (59) with the N-terminal portion of WE14 (WSMRD) (Table 3). This 2.5HIP was also found to be recognized by two other pathogenic CD4^+^ T cell clones from the BDC panel (BDC-9.46 and BDC-10.1) (25). Remarkably, BDC-6.9 and BDC-9.3 were shown to recognize another HIP (designated the 6.9HIP), which also contained LQTLAL, but in this instance fused to the N-terminal part of propeptide 2 (NAARDP) (Table 3), a natural cleavage product of islet amyloid polypeptide (25). These findings suggest that HIP formation and recognition by CD4^+^ T cells are not rare events.

When HIP-reactive BDC clones are adoptively transferred to young (<2 weeks of age) NOD mice, diabetes is dramatically accelerated, demonstrating their pathogenicity (27, 58). Additional observations further support the notion that CD4^+^ T cells responsive to HIPs are important contributors to the disease process. HIP-reactive T cells are among the first CD4^+^ T cells to enter the pancreas of NOD mice, and their numbers increase in both the pancreas and the peripheral blood as the autoimmune process progresses (27). Of importance, the 6.9HIP was recently shown to be naturally presented by H2-A^g7^ class II MHC molecules isolated from both islet APCs and the pancreatic lymph nodes of NOD mice (60), confirming HIPs as natural ligands for class II MHC.

The seminal discovery of HIPs as CD4^+^ T cell epitopes in NOD mice led to the investigation of their relevance to patients with T1D. A peptide library of 16 candidate HIPs was constructed in which the first segment of the hybrid peptide was either the human version of the C-peptide fragment found in the murine HIPs (SLQPLAL) or one of two C-peptide fragments predicted to bind well to the N-terminal half of HLA-DQ8 (GQVELGG or GQVELGGG). The second segment contained human sequences of the N-terminal portions of peptides that are found in mouse beta cell extracts and are natural cleavage products of secretory granule proteins (25, 28). Upon screening of the library with CD4^+^ T cell lines and clones that had been isolated from the islets of deceased T1D donors, four HIPs in the library were identified as T cell epitopes (Table 3) (2, 25). The use of peripheral blood mononuclear cells from patients with T1D led to the identification of a remarkable ten additional HIPs as T cell epitopes (Table 3) (28). Although two of the human HIPs recognized by islet-infiltrating T cells were verified as being recognized in the context of HLA-DQ8 (Table 3), definitive MHC restrictions for several HIPs have not yet been reported.

Recently, a peptide library was designed to specifically identify HIPs recognized in the context of HLA-DR molecules incorporating HLA-DRB1∗04:01 (DR0401) as their beta chain (26). To construct this library, one of 86 proinsulin fragments was combined *in silico* with one of 89 natural cleavage products from secretory granule or otherwise islet-associated proteins. HIP binding to DR0401 was predicted, and binding of the top 50 candidates was then experimentally determined. Thirty of these candidates bound to DR0401 with measurable affinity and were used in pools for *in vitro* stimulation of peripheral blood mononuclear cells from patients with T1D over a 2-week period. DR0401 tetramer staining of the resulting cultures facilitated the identification of six HIPs as T cell epitopes (Table 3) (26). Taken together, the identification of HIP-reactive CD4^+^ T cells in the islets and peripheral blood of patients with T1D marks a satisfying translation from mice to men.

## Noncontiguous CD8^+^ T cell epitopes

The identification of HIPs as targets for CD4^+^ T cells in NOD mice and patients with T1D helped to spark an interest in the search for noncontiguous CD8^+^ T cell epitopes in autoimmune diabetes, a field that is now in its infancy (23, 24). Examples of protein sources for tumor-associated *cis*-spliced peptides include fibroblast growth factor 5 (55), melanocyte protein PMEL (54, 56, 57), and tyrosinase (53); the removed intervening sequence ranges from 2 to 40 amino acids (53, 54, 55, 56, 57). A *cis*-spliced peptide derived from human nuclear autoantigen Sp-100, and acting as a minor histocompatibility antigen, has also been reported (61). Although technical challenges prevented an assessment of the overall occurrence of class I MHC-bound spliced peptides, they were thought to be rare. Recent advances have changed this view and revealed that spliced peptides are common, potentially representing up to one-third of the HLA class I immunopeptidome (62, 63). This observation has greatly stimulated the search for spliced peptides as potential CD8^+^ T cell epitopes in multiple conditions, including cancer (64, 65), infectious diseases (66, 67, 68), and autoimmune diseases such as T1D (23, 24).

To date, two *cis*-spliced peptides derived from beta cell proteins, one from islet amyloid polypeptide and one from receptor-type tyrosine-protein phosphatase-like N, have been reported as T cell epitopes in humans (Table 4) (23, 24). They were identified from the HLA class I immunopeptidome of a human beta cell line by applying reported peptide splicing preferences (69) to ten putative or known beta cell autoantigens and then adding the predicted spliced sequences to the database used for assigning identities to mass spectra (24).

**Table 4:** Noncontiguous CD8^+^ T cell epitopes for islet antigens in humans

Host	Segment 1			Segment 2			IEDB epitope identifier	MHC	T cell source	Reference
	Protein (*gene*)	Position	Sequence	Protein (*gene*)	Position	Sequence				
Human	Islet amyloid polypeptide (*IAPP*)	15–17	VAL	Islet amyloid polypeptide (*IAPP*)	5–10	KLQVFL	952568	A∗02:01	Blood, islets, PaLN	(24)
	Receptor-type tyrosine-protein phosphatase-like N (*PTPRN*)	576–580	SVLLT	Receptor-type tyrosine- protein phosphatase-like N (*PTPRN*)	708–711	RLAK	952407	A∗03:01	Blood	(23)

Abbreviations: IEDB, Immune Epitope Database; PaLN, pancreatic lymph node.

There is emerging evidence that spliced peptides may also be targeted in the NOD mouse. AI4 is a pathogenic CD8^+^ T cell clone isolated from the islets of an NOD mouse (70). By conducting an insulin peptide library screen, we previously found that AI4 recognizes the insulin-derived peptide YQLENYC in the context of H2-D^b^ (71). Identification of this 7-mer peptide was unexpected, as H2-D^b^ favors longer peptides (9mers and 10mers) and those having Leu, Ile, or Met at the C-terminal anchor position (72). However, the Cys in YQLENYC is the penultimate residue encoded by both *Ins1* and *Ins2*, and addition of the last residue (Asn) abolished recognition. Several extensions at the C terminus of YQLENYC were tolerated by AI4 (71), suggesting the potential of spliced peptides to be recognized by pathogenic beta cell-cytotoxic CD8^+^ T cells in T1D and to serve as additional, or perhaps even sole, ligands for them.

## Formation of noncontiguous T cell epitopes

In the T1D sphere, HIPs were reported as T cell epitopes (25) before spliced peptides were (24); however, spliced peptides as epitopes for CD8^+^ T cells in cancer immunology were discovered over a decade before HIPs were described as CD4^+^ T cell targets (55, 57). Thus, the mechanism of formation of spliced peptides will be discussed first, in accordance with the historical order of discovery of these two classes of noncontiguous T cell epitopes.

The proteasome is a multisubunit barrel-shaped cytosolic threonine protease that is the major source of peptides presented by class I MHC (73). The peptides are delivered to the endoplasmic reticulum by the transporter associated with antigen processing, in some cases requiring N-terminal trimming by aminopeptidases in the endoplasmic reticulum before MHC binding (74). Upon protein cleavage by the proteasome, an acyl-protease intermediate is formed in which a fragment of the cleaved protein is linked to the catalytic threonine. This intermediate is usually then hydrolyzed. Early investigations suggested that the proteasome also participated in the generation of *cis*-spliced peptides, as presentation to cognate T cells was blocked by proteasome inhibitors (55, 57, 61). Furthermore, purified 20S proteasomes were capable of generating a *cis*-spliced peptide when incubated with a relevant region of the precursor protein (57, 61). This occurred by transpeptidation in which the protein fragment linked to the catalytic threonine in the acyl-protease intermediate was transferred to another peptide whose N terminus participated in an aminolysis reaction before the normally favored hydrolysis process could occur (57, 61).

Although evidence suggests that noncontiguous CD8^+^ T cell epitopes are generated primarily by proteasome-mediated peptide splicing involving transpeptidation (75), the generation of HIPs remains more of a biochemical mystery to date. The sites of formation of HIPs are also under investigation; this knowledge could shed light on how they are produced. As previously reviewed (76), proteases have the capability to catalyze not only peptide bond hydrolysis but also reverse proteolysis (condensation), depending on the prevailing conditions. Upon their initial discovery, HIPs were proposed to form by reverse proteolysis in the secretory granules of beta cells, the site of proteolytic processing of insulin and multiple other proteins, with the molecular crowding that characterizes the beta cell secretory granules promoting peptide ligation (25). By analogy to spliced peptide formation by the proteasome, secretory granule proteases that employ formation of an acyl-protease intermediate may also participate in HIP formation by transpeptidation. In support of the notion that HIPs form in the beta cell secretory granules, the 6.9HIP was identified by mass spectrometry in the antigenic fractions of secretory granules enriched from NOD mouse-derived beta cell tumors (29). HIPs have also been identified in NOD mouse islet crinosomes (formed by fusion of secretory granules and lysosomes), suggesting they may arise within these structures also (60). One potential mechanism for HIP formation is suggested by the recent report that the lysosomal cysteine protease cathepsin L can create HIPs by transpeptidation when presented with appropriate precursors *in vitro* (77). The elution of the 6.9HIP from H2-A^g7^ class II MHC molecules isolated from islet APCs or pancreatic lymph node cells of NOD mice indicates that non-beta cells can present HIPs to T cells (60). However, whether noncontiguous CD4^+^ T cell epitopes can be generated in cells other than beta cells is unclear at this time. There are at least two potential mechanisms by which APCs could obtain HIPs from beta cells (Fig. 1). First, islet APCs are known to acquire vesicles, including intact secretory granules, from beta cells (78, 79). Second, peptides in crinosomes can be released from beta cells into the circulation and can then be captured and presented by APCs in peripheral lymphoid organs (80) and the blood (81).

## Advances in noncontiguous epitope discovery

T cell epitope discovery has evolved from functional mapping of T cell responses using screening of cDNA or overlapping peptide libraries to more comprehensive approaches involving unbiased mass spectrometry–based discovery tools (82). The identification of spliced peptides as potential class I MHC ligands and CD8^+^ T cell epitopes has undergone a similar evolution. Early studies used a combination of cDNA screening and libraries of overlapping peptides that incorporated deletions of intervening sequences to map the specificity of tumor-reactive CD8^+^ T cells (55, 57). Refinement of this approach to incorporate *in vitro* proteasome digests and mass spectrometry to detect posttranslationally spliced peptides generated through transpeptidation reactions has improved the identification of candidate spliced peptide epitopes (53, 56, 65, 66, 67, 83, 84, 85, 86). Prior knowledge of the input peptide precursors in these *in vitro* digestion experiments constrains the search space for peptide spectral matching to the raw MS/MS data produced during tandem mass spectrometry experiments. However, for a peptide to function as a T cell epitope, it must not only be generated but also bind an MHC molecule, *i.e*., the peptide must be part of the immunopeptidome. Searching global MS/MS data from class I MHC-eluted peptides for spliced peptides is challenging owing to the enormous size of databases required to account for all possible *cis*- and *trans*-spliced peptides. Such databases are computationally prohibitive and their size contributes to high false discovery rates (62, 87). Liepe *et al.* (63) circumvented this issue to some extent by generating a smaller database of *cis*-spliced peptides only and prefiltering the number of spliced peptides searched based on accurate mass of the precursor ions observed in the mass spectrometry experiment. Although this approach enabled global analysis of spliced peptides, and surprisingly showed that up to 30% of HLA class I–bound peptides were potentially spliced in origin, it was still computationally intensive and biased to screening only *cis*-spliced peptides with a relatively short intervening sequence. Faridi *et al.* (62) subsequently used a different approach that harnessed *de novo* sequencing of high-quality MS/MS spectra that were not explained by a templated or linear sequence. This approach essentially used information in the MS/MS spectra to elucidate potential sequences, which were then filtered post analysis using an algorithm called “Hybrid-Finder” that searched for possible spliced explanations of the sequences. These sequences were then appended to the reference proteome to generate a hybrid database containing potential spliced peptides for a subsequent more conventional search of the MS/MS data. Using this approach, these investigators also demonstrated that around 30% of peptides in the immunopeptidome of several HLA class I allotypes were best explained by noncontiguous sequences.

Despite strong functional evidence for their existence, this high proportion of spliced peptides within the immunopeptidome remains contentious. For instance, Mylonas *et al.* (88), using a similar approach to Faridi (62), found that only 2% to 6% of peptides within their datasets were best explained as spliced peptides, although they constrained their searches to *cis*-spliced peptides. Similarly, other investigators have sought alternative explanations for sequences by considering other sources, including translation of novel unannotated, or cryptic, open reading frames (89, 90). Regardless, these represent previously unannotated sources of the immunopeptidome and future mechanistic studies will likely resolve the source of all classes of peptides.

The contribution of noncontiguous peptides to the class II MHC immunopeptidome has received far less attention than that of class I. To date, only the 6.9HIP has been definitively identified as a class II MHC-bound noncontiguous peptide (60). However, proteomic analysis has detected a number of HIPs in both mouse and human islets (91). For these studies, a computer algorithm was used to generate mouse and human HIP databases in which all possible C-terminal truncations of the insulin C-peptide were joined to all natural cleavage products of the secretory granule proteins chromogranin-A, insulin, islet amyloid polypeptide, pro-neuropeptide Y, and secretogranin-1. These potential HIPs were then incorporated into a reference proteome database. This strategy led to the detection of multiple HIPs in both mouse and human islets (91). Vital considerations for the validation of HIPs identified by immunopeptidomic and proteomic analyses have been discussed (60, 91).

## Possible ramifications of beta cell–derived posttranslationally modified T cell epitopes for disease pathogenesis

A proportion of beta cell–reactive T cells evade the central tolerance mechanism of negative selection despite the thymic expression of several important beta cell antigens, including insulin (92). This has been explained in part by the finding that the cognate peptides for these T cells in humans are sometimes characterized by weak MHC binding (93, 94, 95). Poor peptide binding to MHC would lead to a low-avidity interaction between the T cell receptor (TCR) of the developing T cell and peptide/MHC in the thymus, allowing cognate T cells to escape negative selection. Similarly, reduced affinity of the TCR for its cognate peptide/MHC would have a similar outcome. Although low-avidity interactions are satisfying to explain central tolerance evasion, they would seem unfavorable for T cells to cause beta cell destruction. In the case of highly expressed beta cell antigens such as insulin, it is thought that high abundance of weak MHC-binding peptides could lead to sufficient peptide/MHC for T cell recognition in the periphery.

The need for such arguments explains the unique appeal of posttranslationally modified T cell epitopes in general (96), and HIPs in particular. Some posttranslational modifications are increased as a result of endoplasmic reticulum stress in beta cells (17), which results from the tremendous demand for insulin production that is placed on them. They can also be increased upon exposure to inflammation (97). Furthermore, in the case of HIPs in particular, their formation likely requires the conditions uniquely present in beta cell secretory granules and crinosomes (25, 29, 60, 77). Thus, the conditions favoring the formation of posttranslationally modified epitopes would restrict them to production in the target organ and would exclude them from the thymus. Therefore, strong MHC binding and/or high TCR affinity would be possible, presumably facilitating T1D pathogenesis.

It was initially assumed that unique populations of T cells were activated in response to posttranslationally modified peptides and that these T cells would not recognize the unmodified versions. This explains why modified beta cell–derived peptides are often referred to as “neo-epitopes.” However, emerging findings suggest this is not true in all cases. For example, deamidated insulin-derived peptides were recently reported to be presented by class II MHC molecules in NOD mice (60). However, T cells capable of recognizing only the deamidated peptides were not found. Instead, the deamidated peptides exhibited improved MHC binding and enhanced the activation of T cells also reactive to the unmodified peptides (60). This is an important result indicating that unconventional epitopes, including noncontiguous ones, may contribute to T1D pathogenesis through at least two different mechanisms.

## Future directions

HIPs have been detected by mass spectrometry in the islets of NOD mice as well as mice that are not susceptible to autoimmune diabetes (91). HIPs have also been identified in islets from normal human donors (91), suggesting that their mere presence is insufficient to precipitate disease, likely due at least in part to the absence of T1D-predisposing class II MHC molecules to present them to T cells. Quantitative differences between HIPs in normal and T1D-susceptible individuals cannot be excluded and should also be considered. In NOD mice, HIP-reactive T cells have shown promise as markers of ongoing autoimmunity, with the frequency of antigen-experienced T cells increasing with time (27). In humans, HIP-reactive T cells are not limited to at-risk individuals and patients with T1D but are found in class II MHC-matched normal controls as well (26, 28). However, a combined evaluation of T cell number and function for multiple HIP-reactive T cell populations may allow a disease-specific profile to be elucidated (26, 28, 98). Future essential work will ideally uncover HIP reactivities that are more confined to the disease state. Besides serving as disease biomarkers, HIP-reactive T cells may also serve as targets of peptide-based antigen-specific preventive and therapeutic strategies (99). A better understanding of the mechanism(s) of formation of HIPs could present additional interventional opportunities.

Thus far, the identification of HIPs using T cell, proteomic, and immunopeptidomic analyses has taken a largely candidate approach. As bioinformatics and mass spectrometry advances continue to be made, more unbiased strategies for immunopeptidomic analysis will need to be brought to bear on this problem. Such strategies, recently applied to class I MHC immunopeptidomes (62, 63, 64, 65, 82, 88, 89, 100), will also be critical for the unbiased discovery of noncontiguous CD8^+^ T cell epitopes in autoimmune diabetes.

The identification of HIPs as CD4^+^ T cell epitopes in NOD mice quickly translated to the human disease and should serve to reinvigorate interest in the NOD mouse model as an antigen and epitope discovery tool. It is our intention that this review will help to expedite and guide these investigations.

## Conflict of interest

The authors declare that they have no conflicts of interest with the contents of this article.
